# Uncertainty estimation in diagnosis generation from large language models: next-word probability is not pre-test probability

**DOI:** 10.1093/jamiaopen/ooae154

**Published:** 2025-01-10

**Authors:** Yanjun Gao, Skatje Myers, Shan Chen, Dmitriy Dligach, Timothy Miller, Danielle S Bitterman, Guanhua Chen, Anoop Mayampurath, Matthew M Churpek, Majid Afshar

**Affiliations:** Department of Biomedical Informatics, University of Colorado Anschutz Medical Campus, Aurora, CO 80045, United States; Department of Medicine, University of Wisconsin-Madison, Madison, WI 53792, United States; Department of Medicine, University of Wisconsin-Madison, Madison, WI 53792, United States; Artificial Intelligence in Medicine (AIM) Program, Mass General Brigham, Harvard Medical School, Boston, MA 02114, United States; Department of Radiation Oncology, Brigham and Women’s Hospital/Dana-Farber Cancer Institute, Boston, MA 02114, United States; Department of Computer Science, Loyola University Chicago, Chicago, IL 60660, United States; Computational Health Informatics Program, Boston Children’s Hospital, Boston, MA 02115, United States; Artificial Intelligence in Medicine (AIM) Program, Mass General Brigham, Harvard Medical School, Boston, MA 02114, United States; Department of Radiation Oncology, Brigham and Women’s Hospital/Dana-Farber Cancer Institute, Boston, MA 02114, United States; Department of Biostatistics and Medical Informatics, University of Wisconsin-Madison, Madison, WI 53792, United States; Department of Medicine, University of Wisconsin-Madison, Madison, WI 53792, United States; Department of Biostatistics and Medical Informatics, University of Wisconsin-Madison, Madison, WI 53792, United States; Department of Medicine, University of Wisconsin-Madison, Madison, WI 53792, United States; Department of Medicine, University of Wisconsin-Madison, Madison, WI 53792, United States

**Keywords:** large language models, electronic health records, diagnostic uncertainty, machine learning

## Abstract

**Objective:**

To evaluate large language models (LLMs) for pre-test diagnostic probability estimation and compare their uncertainty estimation performance with a traditional machine learning classifier.

**Materials and Methods:**

We assessed 2 instruction-tuned LLMs, Mistral-7B-Instruct and Llama3-70B-chat-hf, on predicting binary outcomes for Sepsis, Arrhythmia, and Congestive Heart Failure (CHF) using electronic health record (EHR) data from 660 patients. Three uncertainty estimation methods—Verbalized Confidence, Token Logits, and LLM Embedding+XGB—were compared against an eXtreme Gradient Boosting (XGB) classifier trained on raw EHR data. Performance metrics included AUROC and Pearson correlation between predicted probabilities.

**Results:**

The XGB classifier outperformed the LLM-based methods across all tasks. LLM Embedding+XGB showed the closest performance to the XGB baseline, while Verbalized Confidence and Token Logits underperformed.

**Discussion:**

These findings, consistent across multiple models and demographic groups, highlight the limitations of current LLMs in providing reliable pre-test probability estimations and underscore the need for improved calibration and bias mitigation strategies. Future work should explore hybrid approaches that integrate LLMs with numerical reasoning modules and calibrated embeddings to enhance diagnostic accuracy and ensure fairer predictions across diverse populations.

**Conclusions:**

LLMs demonstrate potential but currently fall short in estimating diagnostic probabilities compared to traditional machine learning classifiers trained on structured EHR data. Further improvements are needed for reliable clinical use.

## Introduction

**Figure 1.**
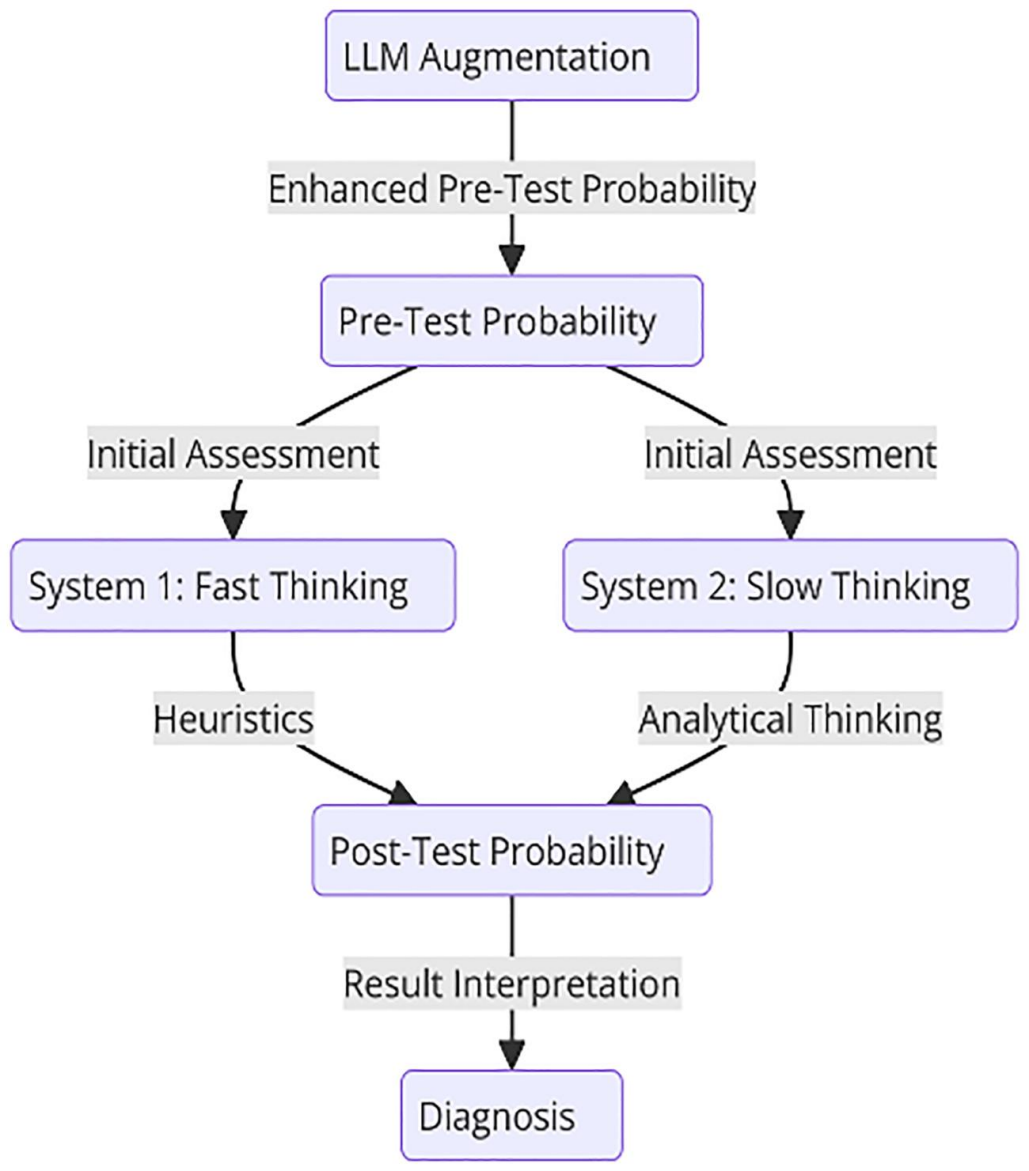
Process map in generating a diagnosis with the role of LLMs to augment human diagnostic reasoning.

Diagnosis in medicine is inherently complex and involves estimating the likelihood of various diseases based on a patient’s presentation. This process requires integrating baseline information to establish pre-test probabilities during the initial hypothesis generation for a diagnosis, followed by iterative refinement as diagnostic test results become available^1^^,^^2^ (Figure 1). Typically, clinicians rely on medical knowledge, pattern recognition and experience, enabling quick hypothesis generation of the initial diagnosis. However, this process is prone to cognitive biases, which can lead to diagnostic errors.^3^ Analytic thinking, a more evidence-based process, is time-consuming and often impractical in fast-paced clinical environments. Although clinicians are taught to estimate a pre-test probability and apply test sensitivity and specificity, cognitive biases and heuristic-based thinking often lead to under- and overestimation of the pre-test probability and subsequent misdiagnoses.^4^^,^^5^

The integration of Large Language Models (LLMs) in diagnostic decision support systems has garnered significant interest in addressing these challenges. Recent advancements, particularly with models like GPT-4, have demonstrated that LLMs can rival clinicians in generating differential diagnoses.^5^^,^^6^ However, LLMs often fail to explicitly convey uncertainty in the estimated probability of a diagnosis in their outputs. This is crucial in medicine; for example, an LLM might suggest an initial diagnosis of pneumonia, yet, a 20% probability of pneumonia may have vastly different implications for a clinician compared to a 90% probability. While GPT-4 has shown some potential for improvement over clinicians in predicting pre-test probability of certain conditions, overall performance is still suboptimal.^4^^,^^5^ LLMs are not designed as classifiers that output probability distributions over specific outcomes; instead, they produce probability distributions over sequences of tokens (words). This raises the research question of how to map these token sequences to clinically meaningful probabilities, particularly for pre-test or post-test diagnosis probability estimation. Addressing this gap is crucial to avoid potential misinterpretations and mitigate the risk of automation bias in clinical settings.

The concept of uncertainty estimation for the generated text in LLMs is rooted in information theory with entropy, which measures the uncertainty of a probabilistic distribution to get next-word prediction. This process involves training the models to align their predictions with the actual distribution of the language they are trained on, resulting in the generation of convincing and coherent natural language. Existing literature investigates methods for extracting uncertainty estimation from LLMs, including token probabilities and verbalized probabilities (confidence).^7^^,^^8^ In particular, Savage et al. 2024 investigates methods of verbalized confidence, sample consistency to assess LLM uncertainty in clinical reasoning tasks using question-answering datasets with open-ended questions.^9^ However, LLMs are known to suffer from the problem of unfaithful generation, where their outputs do not always accurately reflect their underlying knowledge or reasoning.^10^^,^^11^ None of the previous work has compared LLM uncertainty estimation directly with machine learning classifiers, which are trained on structured data, produce calibrated probabilities, and learn explicit relationships between features and outcomes, grounded in the data distribution and class prevalence. Further, while LLMs may have general knowledge about disease prevalence from the pretraining corpora, such as Wikipedia, it remains uncertain whether they can translate general knowledge into patient-specific diagnostic reasoning and estimate pre-test probabilities, a question this paper aims to investigate.

We aimed to address this gap by evaluating the strengths and limitations of LLMs in pre-test diagnostic probability estimation. We conducted a detailed evaluation of 2 open-box LLMs: Mistral-7B-Instruct^12^ and Llama3-70B-chat-hf,^13^ on the task of predicting pre-test diagnostic probabilities. These models were selected because they were available open source and adaptable through instruction-tuning. Unlike previous work exploring LLM medical uncertainty estimation on question-answering tasks or case reports,^3^^,^^10^^,^^14^ our study was based on a set of structured data in the electronic health records (EHRs) from a cohort of 660 patients at a large medical center in the United States. The task involves binary predictions for Sepsis, Arrhythmia, and Congestive Heart Failure (CHF), with positive class distributions of 43%, 16%, and 11%, respectively. Ground truth diagnoses were annotated by expert physicians through chart reviews.^15^ The EHR data included vital signs, lab test results, nurse flow-sheet assessments, and patient demographics. We compared our results to an eXtreme Gradient Boosting (XGB) classifier that used the raw structured EHR data as input, representing the state-of-the-art in many clinical predictive applications.^16^^,^^17^ EHR data included vital signs, structured nurse flowsheet assessments (ie, mental status, mobility, etc.), and lab test results. We subsequently added patient demographics (sex, ethnicity, and race), encoded as categorical variables, to examine if such a setting could improve model performance.

As input to the LLM, we used the same structured data features but transformed them into a textual format, illustrated in the “Methods” section. We examined 3 methods for generating prediction probabilities from LLMs: (1) prompting LLMs to provide verbalized probabilities (*Verbalized Confidence*); (2) using *Token Logits* from a binary Question-Answering (QA) framework (where “A” represents “Yes” and “B” represents “No” to the question of whether a patient has a certain diagnosis); and (3) a feature-based calibrator that takes the embeddings from the LLMs’ last hidden layers as features into an XGB classifier (*LLM Embedding+XGB*). We first evaluated discrimination between classes using the Area Under the Receiver Operating Characteristic curve (AUROC). Then, we reported the Pearson correlation by comparing the positive class predicted probabilities from the LLMs with those predicted by the baseline XGB classifier that was trained on the raw structured data.

## Methods

### Methods of extracting pre-test probabilities from LLMs

This section formulates the task as a binary diagnostic outcome classification using 3 methods. We benchmarked against XGB using raw features (baseline, *Raw Data+XGB*), correlating each method. We utilized a table-to-text method to convert structured EHR data into text, detailed in Gao et al. 2024.^18^ Specifically, we began this transformation by creating a template starting with “Hospitalized patient with age XX, systolic blood pressure YY, …” where XX and YY represent the actual values from patient representation, as presented in Table 1. We then appended each clinical feature, its corresponding value, and its unit of measurement in the text. The study was approved by the University of Wisconsin-Madison (IRB #2019-1258). Source code is available at https://github.com/serenayj/LLM_Embedding_Medical_ML.

**Table 1.** Input EHR variables and table-to-text template used to convert structured EHR data into LLMs.

Input EHR variables	Age, systolic blood pressure, diastolic blood pressure, oxygen saturation, temperature in celsius, proton pump inhibitor, alert, voice, pain, unresponsive scale (AVPU), albumin, alkaline phosphatase, anion gap, total bilirubin, blood urea nitrogen, blood urea nitrogen to creatinine ratio, calcium, chloride, carbon dioxide, creatinine, serum glucose, hemoglobin, platelet count, potassium, serum glutamic-oxaloacetic transaminase, sodium, total protein, white blood cell count
Table-to-text template	Hospitalized patient of age [value] getting worse has labs and vitals values of systolic blood pressure [value] mmHg, diastolic blood pressure [value] mmHg, oxygen saturation[value] %, body temperature [value] celsius degree, … total protein [value], white blood cell [value]. What are the diagnoses for this patient?

Abbreviations: EHR: Electronic Health Records; mmHg: millimeters of mercury.

*Token logits*: We prompted the LLM with a detailed description of the patient’s condition and directly asked for a binary response: “Does the patient have {diagnosis}? A. Yes” or “B. No,” indicating the presence or absence of sepsis. Specifically, the probability estimation was derived from the logits corresponding to these responses. We applied a softmax function yielding a normalized score for each option. We used a zero-shot setting for both LLMs.

*Verbalized confidence*: This approach followed the previous study of GPT-4 on diagnostic probability estimation, and we used the same format of prompt,^4^ posing a more open-ended question to the LLM followed by a narrative description of patient representation: “How likely is it for the patient to have {diagnosis}?” The LLM will provide a percentage score, which we utilized as the probability of positive diagnosis. This approach allowed us to assess the model’s ability to generate and verbalize probability assessments in a natural language format without further binarization of the results. We used zero-setting for both LLMs. Instead of applying a cut-off to categorize the predictions, we evaluated the raw probability estimates directly using AUROC scores and Pearson Correlation.

*Feature-based calibrators*: For the feature-based calibrators, we extracted embeddings from the last hidden states of the Llama3-70B and Mistral-7B models, applying max pooling to generate feature representations for each variable formatted as text input. This process resulted in embedding representations with dimensions of 4096 for Mistral-7B and 8192 for Llama3-70B, respectively. These embeddings were then fed into an XGB classifier. All XGB classifiers were trained using a 5-fold cross-validation on the 660 patient data.

### Prompt development

Our method of prompt development started with a prompt from previous literature that prompts GPT-4 for confidence estimation.^3^ We then made modifications according to the task descriptions and format requirements of the LLMs. To ensure that the LLMs would follow the format requirements, we tested the prompts with a few synthetic examples.

### Training details

All experiments involving XGB classifiers were trained under a 5-fold cross-validation setting. On each fold, we employed grid search for parameters using another 5-fold to select the best hyperparameters.

## Results

**Figure 2.**
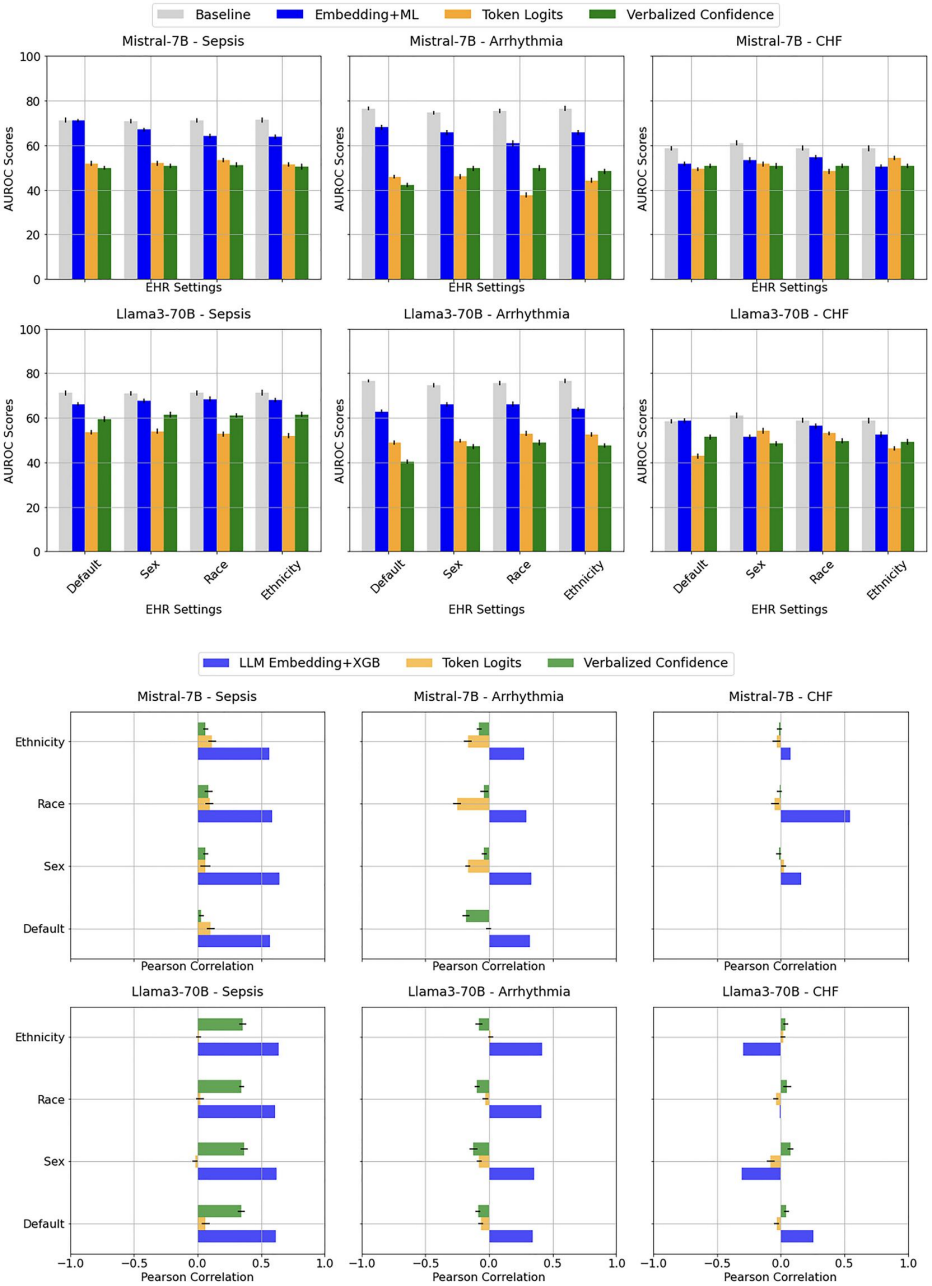
(TOP) Area under the receiver operating characteristic curve (AUROC) scores from both LLMs using different EHR demographics input settings, across the diagnoses prediction of sepsis, arrhythmia, and congestive heart failure (CHF) (BOTTOM) Pearson correlations between the LLM predicted pre-test probabilities and the baseline model (Raw Data+XGB) predicted probabilities.

Figure 2 Top illustrates the results of the AUROC from LLMs to predict Sepsis, Arrhythmia, and CHF. The LLM Embedding+XGB method consistently outperformed the other LLM-based methods. Particularly for Sepsis, it achieved nearly the same AUROC score as the baseline Raw Data+XGB. The Token Logits (mean AUROC: 49.9 with 95% CI: 47.8-51.9) and Verbalized Confidence (mean AUROC: 50.9 with 95% CI: 48.7-53.1) methods exhibited marginal performance, generally not surpassing the baseline XGB classifier. The inclusion of demographic variables (sex, race, ethnicity) changed the AUROC scores, for instance, by as much as 7.22 for Mistral embedding on Sepsis prediction (71.1 on default setting versus 63.9 on ethnicity). However, the direction and consistency of these changes varied depending on the specific context and data included.

Figure 2 Bottom reports the Pearson correlation coefficients between the predicted probabilities from LLM-based uncertainty estimation methods and those from the XGB classifier for 3 diagnoses across different demographics. When correlating the LLMs’ positive class probabilities with the baseline results, the token logits and verbalized confidence methods had more variable correlations, often no correlation or negative correlation, suggesting less alignment with the baseline XGB predicted probabilities. On the contrary, the LLM Embedding+XGB method consistently showed strong positive correlations across all tasks and settings with the baseline XGB classifier, indicating its predictions were closely aligned with the baseline XGB classifier.

**Figure 3.**
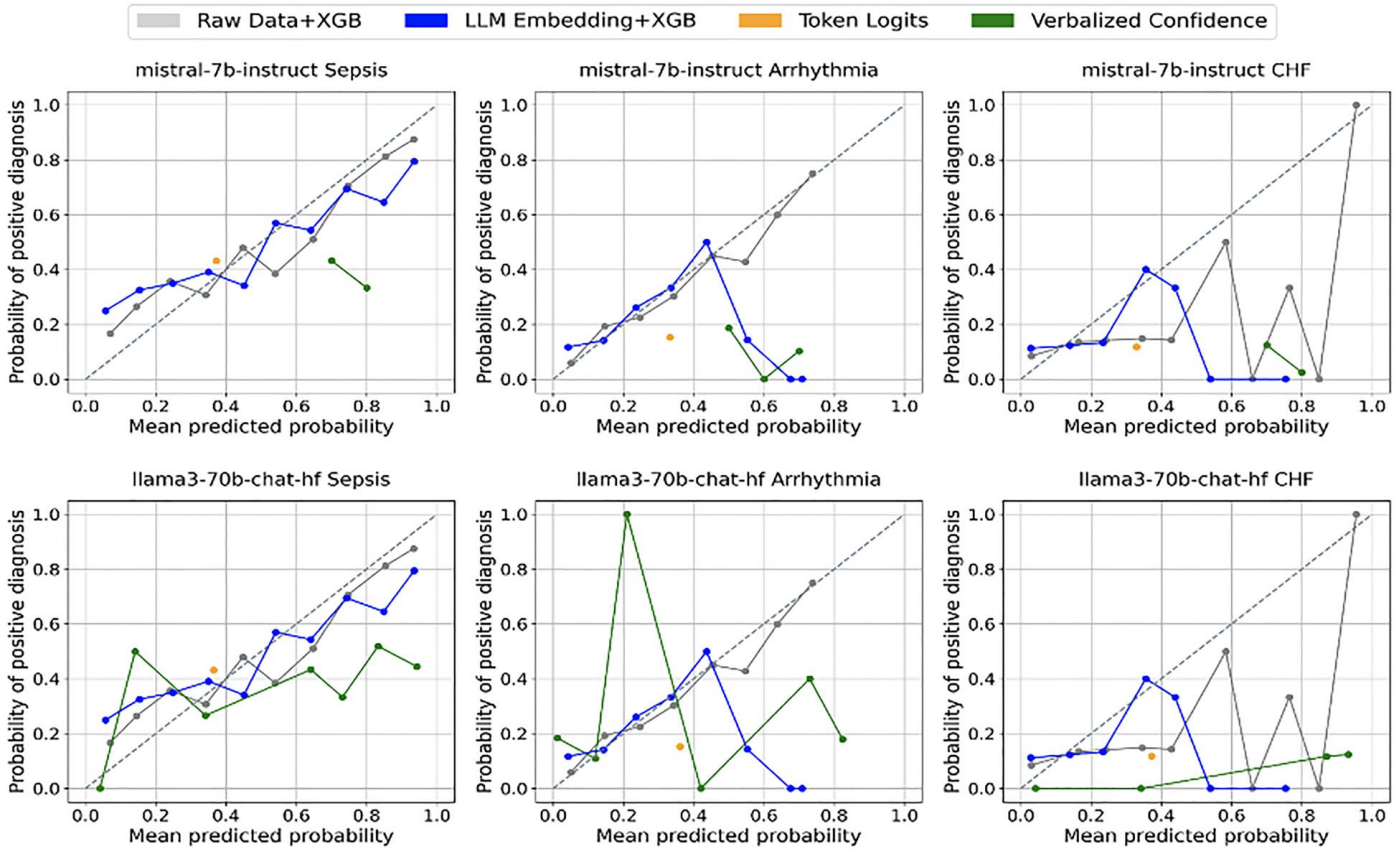
Calibration curves on the 4 probability estimation methods, using Mistral-7B-Instruct and Llama3-70B-Chat-hf on Default EHR setting.

Figure 3 presents the calibration curve of all models on the default setting (no demographic variable). Poor calibration was observed, especially from Token Logits and Verbalized Confidence. Probabilities predicted by the Token Logits always fell between ranges of 0.323 and 0.337. Table 2 further highlights the Expected Calibration Scores (ECE) scores of each method, with notable differences observed across the various biases and diagnoses. For instance, the Verbalized Confidence (Verb. Conf) method tends to exhibit higher ECE values, indicating poorer calibration, especially in the CHF prediction task, while Raw+XGB generally shows more consistent performance across different demographic settings.

**Table 2.** Expected calibration scores (ECE) results across the 4 methods for pre-test probabilities estimation methods, over 3 diagnosis prediction tasks with patient demographic settings.

Method	None	Sex	Race	Ethnicity
	Sepsis, Arrhythmia, CHF	Sepsis, Arrhythmia, CHF	Sepsis, Arrhythmia, CHF	Sepsis, Arrhythmia, CHF
Baseline				
Raw**+**XGB	0.09, 0.02, 0.10	0.07, 0.02, 0.10	0.09, 0.02, 0.10	0.10, 0.02, 0.11
Mistral-7B-Instruct				
Token Logits	0.07, 0.18, 0.21	0.13, 0.21, 0.25	0.13, 0.21, 0.25	0.13, 0.21, 0.25
Verbalized Confidence	0.27, 0.43, 0.59	0.31, 0.35, 0.58	0.27, 0.35, 0.59	0.27, 0.36, 0.58
Embedding**+**XGB	0.09, 0.06, 0.13	0.14, 0.09, 0.09	0.11, 0.11, 0.11	0.06, 0.06, 0.11
Llama3-70B-Chat				
Token Logits	0.07, 0.21, 0.25	0.06, 0.22, 0.25	0.07, 0.21, 0.25	0.06, 0.22, 0.25
Verbalized Confidence	0.28, 0.25, 0.77	0.24, 0.04, 0.01	0.24, 0.04, 0.04	0.24, 0.04, 0.03
Embedding**+**XGB	0.11, 0.05, 0.07	0.06, 0.09, 0.17	0.06, 0.04, 0.09	0.06, 0.06, 0.11

Abbreviations: CHF: Congestive Heart Failure; XGB: eXtreme Gradient Boosting.

### Discussion

These results reflect the lack of robustness of these methods for uncertainty estimation, highlighting a significant gap in uncertainty estimation for medical decision-making. Although these methods are used in literature to assess uncertainty in predicting the next word,^17^ predicting pre-test probabilities requires an understanding of risk based on real-world patient data and disease prevalence, knowledge that LLMs often lack. The introduction of demographic variables complicates the predictive power of these models further due to inherent biases present in LLMs, which may not be trained on a diverse set of patient characteristics, making them susceptible to social biases.

The LLM Embedding+XGB method demonstrated competitive performance compared to the state-of-the-art XGB baseline classifier under specific conditions, such as Sepsis, and exhibited the strongest correlation among the methods tested. However, this result is not surprising given that both methods rely on training a classifier. In contrast, purely LLM-based methods, such as Token Logits (next-word probability) and Verbalized Confidence, were found to be unreliable for risk estimation. Their performance, evaluated through AUROC scores, Pearson Correlation, and calibration curves, deteriorated significantly when diagnosing conditions with lower prevalence, raising concerns about the accuracy of pre-test probabilities derived from these models. The results were consistent across both the Mistral-7B and Llama3-70B models. Additionally, results varied with different demographic characteristics, reinforcing existing concerns about bias in LLMs.^19^ While the LLM Embedding+XGB method showed promise in generating pre-test probabilities, overall, LLM-based probability estimation methods did not achieve the same level of performance as raw tabular data in an XGB model. This underscores the necessity for further optimization of LLM methods to produce uncertainty estimations that align more closely with established and reliable methods.

## Conclusion

Overall, our findings demonstrate the inability of LLMs to provide reliable pre-test probability estimations for specific diseases and highlight the need for improved strategies to incorporate numeracy into diagnostic decision support systems and reduce the impact of bias on LLM performance. This remains a major gap to fill before we can enter a new era of diagnostic systems that integrate LLMs to augment healthcare providers in their diagnostic reasoning.

## Data Availability

The source code and data underlying this article are available in https://github.com/serenayj/LLM_Embedding_Medical_ML. The EHR data underlying this article cannot be shared publicly as it contains protected health information.
